# Information-Theoretic and Conceptual Density Functional Theory Insights on Frustration in Molecular Clusters

**DOI:** 10.3390/e28020213

**Published:** 2026-02-12

**Authors:** Xinyue Zhao, Ziqing Yan, Lei Zeng, Yaqin Zheng, Chunying Rong

**Affiliations:** 1 Key Laboratory of Chemical Biology and Traditional Chinese Medicine Research (Ministry of Education of China), Hunan Normal University, Changsha 410081, China; 2 School of Biological Sciences and Technology, Beijing Forestry University, Beijing 100083, China

**Keywords:** frustration, information-theoretic approach, conceptual density functional theory, molecular complex, energy decomposition, cooperation

## Abstract

Frustration is an intrinsic feature of molecular complexes, arising when individual constituents must distort from their optimal isolated geometries to achieve collective stabilization. Although energetic frustration can be defined as the average distortion energy associated with complex formation, its quantitative origin and its connection to other molecular descriptors remain insufficiently understood. In this work, we systematically investigate frustration in four representative molecular complexes—two homogeneous clusters, (H_2_O)_n_ and (HF)_n_, and two charged clusters, H_3_O^+^(H_2_O)_n_ and F^−^(H_2_O)_n_ (*n* = 1–20)—using three complementary density-based frameworks: (i) total-energy decomposition, (ii) global conceptual DFT (CDFT) descriptors, and (iii) information-theoretic approach (ITA) quantities. Strong linear correlations between the total frustration energy and most energy components, as well as CDFT indices, are revealed, enabling a quantitative interpretation of frustration from energetic and electronic-structure perspectives. Among ITA measures, only a subset, including Shannon entropy, Ghosh–Berkowitz–Parr entropy, Rényi entropy, and the relative Fisher information, exhibits robust and consistent correlations with frustration across all systems, indicating their suitability as ITA-based frustration descriptors. Particularly, the (HF)_n_ clusters show uniformly excellent correlations for all descriptors due to their structurally simple and homogeneous hydrogen-bonding environment. Overall, this work provides a comprehensive density-based understanding of frustration and clarifies which descriptors reliably track its behavior. These insights establish a foundation for applying ITA and CDFT analyses to frustrated phenomena in broader chemical contexts, which could be applied to other systems, including molecular recognition, conformational dynamics, and catalysis.

## 1. Introduction

Molecular complexes stabilized by non-covalent interactions [1] play essential roles across supramolecular chemistry [2], materials science, and biological systems [3]. Proteins, nucleic acids, and molecular assemblies exemplify architectures in which van der Waals forces [4,5,6], hydrogen bonding [7,8], electrostatics [9], and other non-covalent interactions [1,10,11] operate simultaneously. A characteristic feature of such systems is their intrinsic non-additivity: the properties of the whole cannot be obtained by a simple summation of the isolated components. Understanding this emergent behavior requires analyses that can connect global stability with local structural and energetic responses.

From a holistic perspective, cooperativity [12,13,14] reflects the collective “gain” achieved when individual units assemble into a more stable complex. In contrast, at the level of the individual components, frustration [15,16] captures the corresponding “loss”—the energetic penalty each building block incurs when it deviates from its optimal isolated geometry to accommodate the global organization of the complex. These two concepts are complementary: cooperativity describes the stabilization of the whole, whereas frustration quantifies the distortion cost borne by the parts. They are like two sides of the same coin. Together, they provide a dual viewpoint for interpreting the organization and behavior of molecular complexes [17].

Our recent studies have shown that cooperativity [14,18,19,20], defined through building-block interaction energies, can be both positive and negative [12,21,22] and correlates strongly with a variety of density-based descriptors, including energy components [23], information-theoretic approach (ITA) quantities [24,25], and thermodynamic parameters. These results offer valuable insights into the origin of cooperative phenomena [14,19]. In contrast, frustration has thus far been quantified only from a purely energetic standpoint—namely, the average distortion energy per building block. This raises two key questions. Can frustration also be characterized through alternative molecular descriptors, such as conceptual DFT (CDFT) [26] descriptors or ITA quantities? [27,28] And if so, how does energy-derived frustration correlate with these distinct viewpoints?

To address these open questions, we systematically examine four representative molecular complexes, namely, two neutral homogeneous clusters, (H_2_O)_n_ and (HF)_n_, and two charged clusters, H_3_O^+^(H_2_O)_n_ and F^−^(H_2_O)_n_, with n = 1–20. These systems span diverse interaction types and structural features, enabling a broad assessment of frustration in molecular assemblies. We analyze frustration [15,29] using three complementary classes of descriptors: (i) energy components from two total-energy decomposition schemes [30]; (ii) global CDFT indices such as hardness, chemical potential, and electrophilicity [17,31]; and (iii) ITA indices, including Shannon entropy, Fisher information, and related measures [32].

By comparing frustration [33,34,35] derived from total energies with frustration inferred from these alternative descriptors, we aim to clarify which quantities reliably reflect frustrated behavior and what physical insights they provide. This comprehensive investigation establishes a multi-perspective, density-based understanding of frustration and sheds light on its fundamental origin in molecular complex systems.

## 2. Theoretical Framework

Frustration arises when individual components of a molecular complex are forced to deviate from their optimal isolated-state geometries in order to participate in non-covalent assembly. Consider a complex of general composition AB_n_, where A is an optional accessory unit with geometry $R_{A}$, B is the repeating building block with geometry $R_{B}$, and n is the number of such blocks. The optimal isolated structures of A and B, denoted by $R_{0A}$ and $R_{0B}$, serve as their reference states. Upon complex formation, both $R_{A}$ and $R_{B}$ adopt distorted geometries that differ from $R_{0A}$ and $R_{0B}$, placing each component in a frustrated state. In our previous work [13,16], we quantified this phenomenon by evaluating the energetic penalty associated with these distortions and used it to define the degree of frustration [15], or frustrativity, for each building block and for the complex as a whole. The energy sum of the individual components in the formed molecular complex [36] is as follows:

(1) $$E(R)=E_{A}(R_{A})+\sum_{B=1}^{n}E_{B}{(R}_{B}).$$

The energy sum of the individual parts in the reference state is determined as follows:

(2) $$E_{0}(R_{0})=E_{A}(R_{0A})+nE_{B}(R_{0B}).$$

The total frustration energy E_frust_, as a measure of the frustration for the entire system, can be defined as the difference between Equations (1) and (2) [16],

(3) $$E_{frust}=E(R){-E}_{0}(R_{0})>0.$$

This definition rests on the assumption that the total frustration energy reflects the cumulative cost each component incurs by deviating from its optimal isolated state during complex formation. Although the individual parts “sacrifice” their own stability through these distortions, the system as a whole becomes more stable, resulting in an overall energetic gain from the collective perspective. With Equation (3), the frustration energy per building block E_0n_ can be defined as

(4) $$E_{0n}=E_{frust}/n=[E(R)-E_{0}(R_{0})]/n>0.$$

When more repeating blocks are added to the system, the change in the frustration energy per building block may or may not always follow the same trend. To quantify the differences in such trends, we define frustrativity as the partial derivative of E_0n_ with respect to the number of building blocks [17],

(5) $${\zeta =\partial E}_{0n}/\partial n,$$

whose three distinct behaviors are anticipated:

(6) $$\left\{\begin{array}{c} \;\zeta >0,\;\;\;\;\;positively\;frustrative\\ \zeta =0,\;\;\;\;\;\;\;\;\;\;\;non\;frustratative\\ \zeta <0,\;\;\;\;\;egatively\;frustrative \end{array}\right.$$

The behavior of frustration [15,34] can be visualized through the frustrativity profile, which is the variation in the frustration energy per building block, $E_{0n}$, as a function of the number of units n. If adding more units causes $E_{0n}$ to increase, the profile rises, indicating positive frustrativity. Conversely, if additional units lead to a decrease in $E_{0n}$, the profile descends, signaling negative frustrativity. From an energy viewpoint, both types of frustrativity have been observed.

To pinpoint the origin of frustrativity, we employ three approaches in density-based reactivity theory, including two total energy partition schemes [37,38,39,40] from orbital-free DFT (OF-DFT) [30], global descriptors from CDFT [41,42,43], and several ITA quantities [24,25]. In OF-DFT [44], the total energy can be partitioned in two ways. One via

(7) $$E[\rho ]=T_{s}[\rho ]+E_{xc}[\rho ]+E_{e}[\rho ],$$

where non-interesting kinetic energy T_S_, exchange–correlation interaction E_XC_, and total electrostatic energy E_e_ components are employed, and the other through

(8) $$E[\rho ]=E_{s}[\rho ]+E_{q}[\rho ]+E_{e}[\rho ],$$

with E_S_ as the steric energy and E_q_ as the quantum energy [23]. We have shown that

(9) $$E_{s}[\rho ]=T_{w}[\rho ]=\frac{1}{8}I_{F},$$

where T_w_ is the Weizsäcker kinetic energy density functional [45], only different from Fisher information I_F_ by a factor of 1/8 in ITA [28], which will be discussed below. We also have

(10) $$E_{q}[\rho ]=T_{s}[\rho ]+E_{xc}[\rho ]-E_{s}[\rho ].$$

Another framework to appreciate frustrativity is the global descriptors from CDFT. Chemical potential μ (minus of electronegativity χ) and hardness η [46,47] could be obtained from the energies of frontier molecular orbitals, $\epsilon_{HOMO}$ and $\epsilon_{LUMO}$, under the frozen orbital approximation,

(11) $$\mu =-\chi =\frac{\epsilon_{HOMO}+\epsilon_{LUMO}}{2},$$

(12) $$\eta =\epsilon_{LUMO}-\epsilon_{HOMO}.$$

With Equations (9) and (10), the electrophilicity index, ω, is proposed accordingly as [48,49,50]:

(13) $$\omega =\frac{\mu^{2}}{\eta }.$$

The third framework is ITA, where we can leverage different ITA quantities to understand frustrativity, including Shannon entropy S_S_, Fisher information I_F_, Ghosh–Berkowitz–Parr entropy S_GBP_, information gain I_G_, and Rényi entropy R_n_ [25,27,28]. These quantities employ simple density functionals to quantify and appreciate molecular properties such as bonding, stability, and reactivity. They are gauges of different distribution properties of the electron density for the systems. In ITA, Shannon entropy measures the homogeneity of the spatial delocalization of the electronic density, defined as [51,52,53]

(14) $$S_{S}=-\int \rho (r)ln\;\rho (r)dr,$$

whereas Fisher information gauges the sharpness or concentration of the electron density distribution, defined as follows [54,55]:

(15) $$I_{F}=\int \frac{|\nabla \rho (r){|}^{2}}{\rho (r)}dr$$

There is an equivalent formulation of $I_{F}$, called the alternative Fisher information $I_{F}^{\prime }$, whose value is the same as $I_{F}$, but local behavior is vastly different [56]:

(16) $$I_{F}^{\prime }=-\int \nabla^{2}\rho \left(r\right)\ln\rho \left(r\right)dr.$$

The Ghosh–Berkowitz–Parr (GBP) entropy was obtained from transcribing the ground-state density functional theory into a local thermodynamics through a phase-space distribution function. It is defined as follows [57,58]:

(17) $$S_{GBP}=\int \frac{3}{2}k\rho (r)\left[c+ln\frac{t(r;\rho )}{t_{TF}(r;\rho )}\right]dr,$$

where t(**r**;ρ) is the kinetic energy density, t_TF_(**r**;ρ) = cKρ^5/3^(**r**) is the Thomas–Fermi kinetic energy density, and c and k are constants. Another ITA quantity studied in this work is the Rényi entropy of order n, which is defined as [59]

(18) $$R_{n}=\frac{1}{1-n}ln\left[\int \rho (r{)}^{n}dr\right],$$

where n≥0 and n≠1.

Closely related to these ITA descriptors [32] are their relative quantities, which are non-symmetric measures of the difference between two probability distribution functions. These relative ITA quantities include the relative Shannon entropy, also called information gain I_G_ [60,61]; Kullback–Leibler divergence or information divergence, defined as

(19) $$I_{G}=\int \rho (r)ln\frac{\rho (r)}{\rho_{0}(r)}dr;$$

the relative Fisher information [62,63],

(20) G3=IFr=∫ρ(r)∇lnρ(r)ρ0(r)2dr;

the alternative relative Fisher information [64], defined as

(21) G1=IF′r=∫∇2ρ(r)lnρ(r)ρ0(r)dr

(22) $$G_{2}=\int \rho (r)[\frac{\nabla^{2}\rho (r)}{\rho (r)}-\frac{\nabla^{2}\rho_{0}(r)}{\rho_{0}(r)}]dr;$$

and the relative Rényi entropy of order n [65], defined as

(23) Rnr=1n−1ln∫ρn(r)ρ0n−1(r)dr,

where $\rho_{0}(r)$ is the reference electron density satisfying the same normalization condition as ρ(r). Employing all the above indices, the definitions of frustration are the same as Equation (3).

(24) $$Q_{0n}=Q_{frust}/n=[Q(R)-Q_{0}(R_{0})]/n$$

where Q is the quantity from energy partition, CDFT or ITA. We anticipate that these frustrations from DBRT frameworks [17,26] should be able to describe the frustrativity of the molecular complex composed of multiple building blocks. The correlation between energetic frustration and frustration derived from other quantities will be investigated, aiming to elucidate the nature and origin of frustration.

## 3. Computational Details

**Scheme 1:**
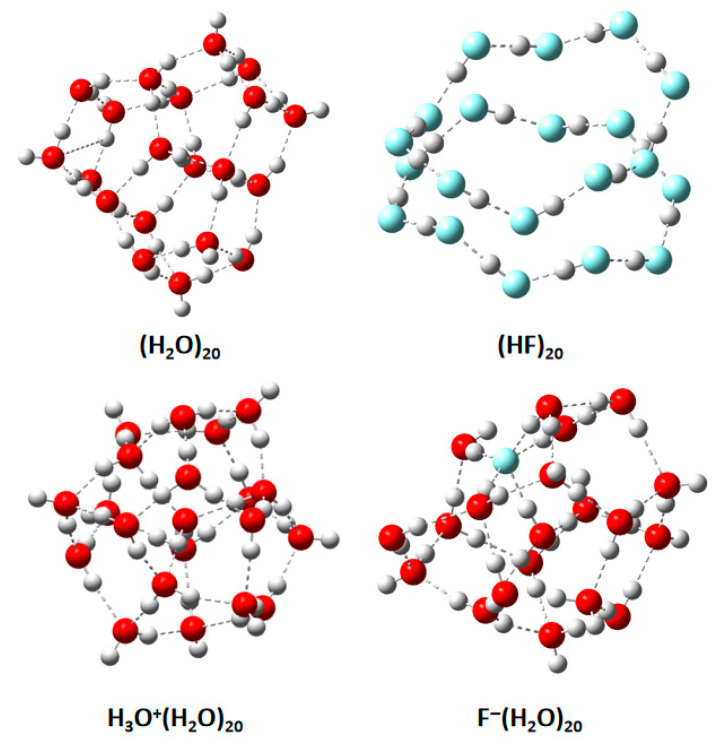
Illustrative examples of the optimized structures of the four cluster models with n = 20.

In this work, we quantify frustrativity using Equation (24) and examine its origin across four representative molecular complex systems, namely, two neutral homogeneous clusters, $\left(H_{2}O{)}_{n}\right.$ and $\left(HF{)}_{n}\right.$, and two charged clusters, ${H_{3}O^{+}(H_{2}O)}_{n}$ and ${F^{-}(H_{2}O)}_{n}$, with repeating blocks n=1–20. Initial cluster geometries, corresponding to the reported global minima for each size, were taken from our previous studies [16,17]. Representative optimized structures [66] for the n=20 clusters are shown in Scheme 1.

All structural optimizations and electronic-structure calculations were performed using Gaussian 16 (Revision A.03) [67]. The isolated building blocks (H_2_O, HF, and H_3_O^+^) and all cluster structures were optimized at the M06-2X/6-311+G(d,p) level of theory [68,69]. While range-separated functionals are increasingly used for extended systems, M06-2X has been shown to provide structural accuracy for H-bonded clusters comparable to RSH functionals and high-level ab initio methods [69,70,71,72]. Benchmark tests with different functionals, including B3LYP-D3(BJ), M062X-D3, ωB97XD, BLYP-D3(BJ), and CAM-B3LYP-D3(BJ), were systematically conducted, and qualitatively, no different results or conclusions were obtained, for which the results are listed in Supplementary Materials (Tables S1–S5). The trends and correlations emphasized in this work are robust across the tested functionals.

Single-point energy calculations for both isolated and distorted (frustrated) components were carried out at the same level to ensure consistency. Energy components were extracted from Gaussian using the keyword iop(5/33 = 1). The steric energy [23] was evaluated from Fisher information [73], which is eight times the corresponding Weizsäcker kinetic energy.

Electron density analyses were performed with Multiwfn 3.8 using Gaussian-generated checkpoint files [74]. A full set of ITA quantities [25,28,32], including those derived from energy density, was computed for each individual molecule. Global CDFT descriptors, including $\varepsilon_{HOMO},\;\;\varepsilon_{LUMO},\;\;\eta ,\;\;\mu ,$ and ω [46,47,49], were obtained directly from Gaussian single-point calculations. All computed quantities were then used to evaluate their corresponding frustration properties for each cluster size.

## 4. Results and Discussion

### 4.1. Frustrativity Profiles from Total Energies

**Figure 1:**
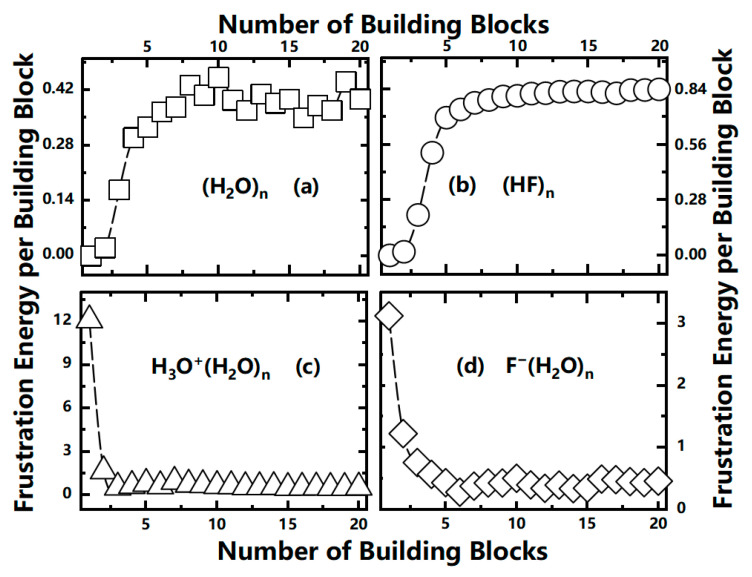
Frustration profiles for the four systems studied in this work as a function of the number of building blocks. Cases (**a**,**b**) display positive frustration, and cases (**c**,**d**) exhibit negative frustration. Units of the frustration profile are in kcal/mol.

Figure 1 illustrates the frustration profiles of the four molecular systems, expressed as the frustration energy per building block $E_{0n}$ (Equation (3)) as a function of cluster size n. For the two homogeneous systems [75], $\left(H_{2}O{)}_{n}\right.\;$ and $\left(HF{)}_{n}\right.$, $E_{0n}$ increases monotonically with n, indicating positive frustrativity. As more monomers join the cluster, each unit must undergo greater average distortion from its optimal isolated geometry, thus sacrificing more individual stability.

In contrast, the two charged systems [76] ${H_{3}O^{+}(H_{2}O)}_{n}$ and ${F^{-}(H_{2}O)}_{n}$ exhibit negative frustrativity: the addition of new units reduces the average distortion penalty. This behavior is consistent with previous literature and reflects the stabilizing influence of charge–dipole [77] and charge-assisted hydrogen bonding [78], which allows structural distortions to be distributed more favorably as the cluster grows. Importantly, frustrativity does not correlate simply with whether a system is neutral or charged, as counterexamples do exist, highlighting that the sign of frustrativity is a property emergent from structural organization rather than overall charge. These results establish the energetic baseline against which all density-based frustration descriptors [43] are compared below.

### 4.2. Energy-Decomposition Analysis: Origins of Energetic Frustration

**Figure 2:**
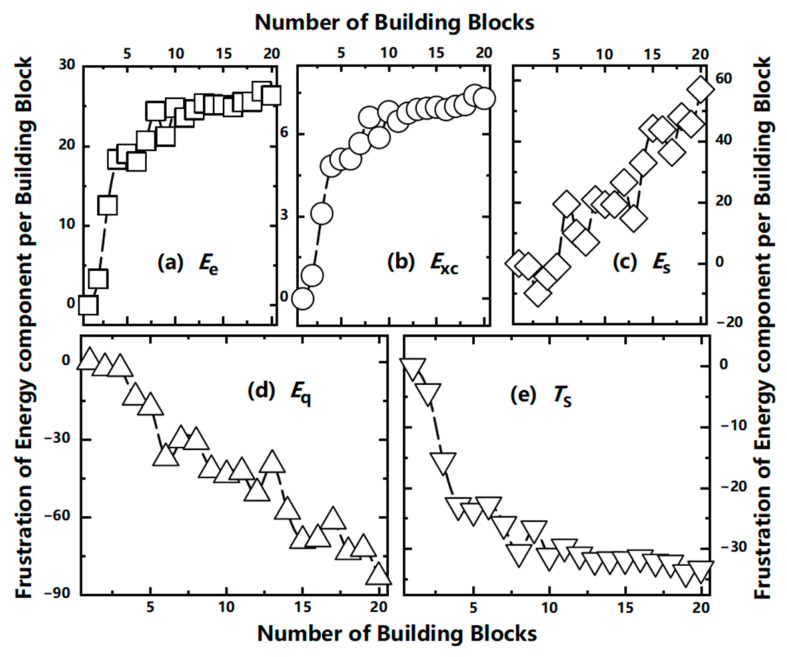
Five energy components from two energy partition schemes for the neutral water cluster in this work as a function of the number of building blocks: (**a**) electrostatic energy E_e_; (**b**) exchange–correlation energy E_XC_; (**c**) steric energy E_S_; (**d**) quantum energy E_q_; (**e**) kinetic energy T_S_. Units of the energy components profile are in kcal/mol.

To understand the components underlying energetic frustration, we evaluated frustration profiles for five energy terms derived from two partitioning schemes (Equations (7) and (8)) [23,38]. Figure 2 shows these profiles for $\left(H_{2}O{)}_{n}\right.$.

For the water cluster, the frustration in the electrostatic energy $E_{e}$, exchange–correlation energy $E_{xc}$, and steric energy $E_{s}$ all increase with cluster size, mirroring the trend of total energy frustration. These descriptors, therefore, exhibit positive frustrativity, with high linear correlations to $E_{0n}$. In contrast, the non-interacting kinetic energy $T_{s}$ and quantum energy $E_{q}$ display negative trends. This sign reversal aligns with the mathematical relationships among the components: $E_{q}$ contains a kinetic contribution subtracted from $E_{xc}$, while $E_{s}$ is directly proportional to the Weizsäcker kinetic term. These opposite trends highlight the subtle balance between different energy components when molecular fragments distort in clusters.

Table 1 summarizes the correlation coefficients for all four systems. Regardless of cluster type, $T_{s}$, $E_{e}$, and $E_{xc,}$ from Equation (7) correlate strongly with the total frustration energy (|R| > 0.91). The steric and quantum energies from Equation (8) show more system-dependent behavior, including sign inconsistencies across the four complexes. The strong and positive correlation with $E_{e}$, which is the only common term in Equations (7) and (8), agrees well with our results for cooperativity [12,13,21,22], indicating that the dominant contributor of frustrativity is the long-range, electrostatic interaction. Steric and exchange–correlation effects may play an indispensable role, but their roles are minor.

An especially noteworthy case is $\left(HF{)}_{n}\right.$, which shows near-perfect correlations for all energy components. We attribute this to the simple, unidirectional hydrogen-bond network and the single structural degree of freedom (bond length) in the HF monomer [79]. This structural uniformity leads to highly coherent distortion patterns across the cluster, producing clean, nearly linear frustrated trends in all energy descriptors.

### 4.3. Frustration from CDFT Descriptors

**Figure 3:**
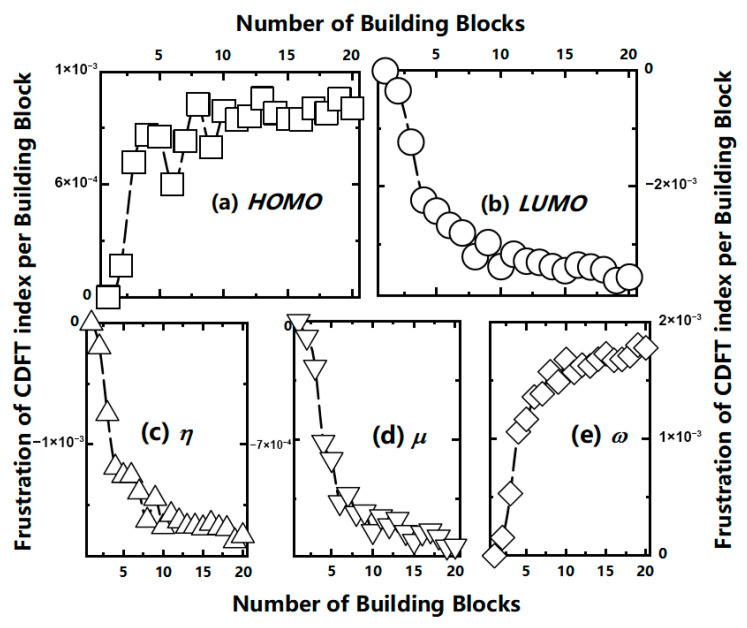
Five CDFT indices for the neutral water cluster in this work as a function of the number of building blocks: (**a**) $\epsilon_{HOMO}$; (**b**) $\epsilon_{LUMO}$; (**c**) chemical hardness η; (**d**) chemical potential μ; (**e**) electrophilic index ω.

Figure 3 shows the frustration profiles of five global CDFT descriptors for $\left(H_{2}O{)}_{n}\right.$: $\varepsilon_{HOMO},{\;\varepsilon }_{LUMO},\;\;\eta ,\;\;\mu ,$ and ω. The HOMO energy and electrophilicity index ω exhibit positive trends (increasing frustration with cluster size), while the LUMO energy, hardness η, and chemical potential μ exhibit negative trends. We also present the frustration plots of CDFT descriptors for $\left(H_{2}O{)}_{n}\right.$ obtained with the ωB97XD functional (Figure S1), as well as the plots for ${H_{3}O^{+}(H_{2}O)}_{n}$ calculated using two different functionals as examples (Figures S2 and S3) in the Supplementary Materials. The frustration behaviors of the global CDFT descriptors are analogous for different functionals and demonstrate that they are insensitive to the choice of different yet similar functionals.

As summarized in Table 2, these sign patterns are consistent across nearly all four molecular systems, with two minor exceptions: the chemical potential frustration in ${H_{3}O^{+}(H_{2}O)}_{n}$ and the HOMO frustration in ${F^{-}(H_{2}O)}_{n}$. These two exceptions persist across all the tested functionals, with only minor differences in the magnitude of their linear correlations (listed in Tables S2 and S3). We hypothesize that these exceptions arise from the extra charges in the molecular complexes, yet elucidating their underlying causes requires further in-depth study, which is outside the scope of this work. Fortunately, the signs of these correlations remain entirely consistent across all functional tests, and thus these two exceptions do not affect the main conclusions of this work. Importantly, the magnitude of the linear correlations is uniformly high for all descriptors across all four complexes, demonstrating that CDFT global measures respond strongly and systematically to geometric distortions associated with frustration.

The observed sign behavior carries intuitive physical meaning. Increasing frustration (higher distortion energy) corresponds to higher HOMO energies and a higher electrophilicity index ω, reflecting enhanced electron-donating and electron-accepting tendencies under structural strain. Conversely, the decreasing chemical hardness and chemical potential reflect a reduced resistance to stabilization mechanisms [80] as clusters deform. Together, these results show that CDFT descriptors provide a reliable and physically interpretable description of frustration. However, it should be noted that Koopmans’ theorem, namely, the frozen orbital approximation (Equations (11)–(13)), neglects such physical effects as electron relaxation, electron correlation, orbital polarization, and basis set incompleteness, and is only applicable to a limited range of closed-shell neutral molecular systems.

### 4.4. ITA Descriptors of Frustration

The third viewpoint employs electron-density distribution measures from ITA. Table 3 reports the correlation coefficients (R) and probability values (*p*-values) between ITA-based frustrations and total energetic frustration for the four molecular systems.

From the data in Table 3, only four ITA descriptors, i.e., Shannon entropy $S_{S}$, Ghosh–Berkowitz–Parr entropy $S_{GBP}$, second-order Rényi entropy $R_{2}$, and the relative Fisher information $G_{3}$, show consistent correlation signs across all systems. These quantities therefore emerge as the most reliable ITA-based fingerprints of frustration. To demonstrate the functional robustness of such reliability, Table S6 in the Supplementary Materials lists the correlation coefficients between these four ITA quantities and the energy frustration for neutral water clusters as an example at the ωB97XD functional level. These consistently signed and strongly significant correlation coefficients indicate that the behaviors of these key ITA quantities are free of functional dependence. In contrast, however, the other ITA-derived indices do not exhibit consistent and significant linear correlations.

**Figure 4:**
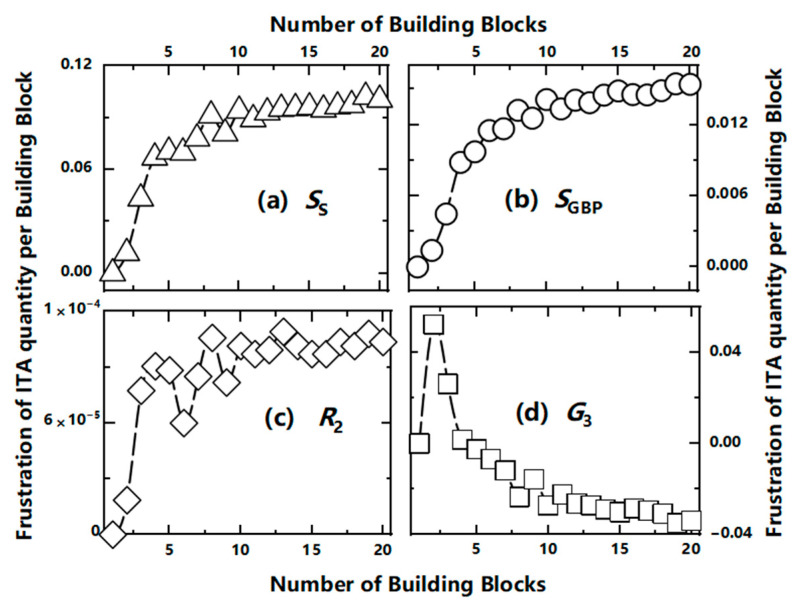
Four information-theoretic quantities for the neutral water cluster in this work as a function of the number of building blocks: (**a**) Shannon entropy S_S_; (**b**) Ghosh–Berkowitz–Parr entropy S_GBP;_ (**c**) Rényi entropy of the second order R_2_; (**d**) relative Fisher information G_3_.

For $\left(H_{2}O{)}_{n}\right.$, Figure 4 illustrates the frustration profiles of these four descriptors. The frustration trends for $S_{S}$ and $S_{GBP}$ are smooth and monotonic, resembling those of total energy frustration. In contrast, some other ITA descriptors, e.g., $G_{1}$ or higher-order Rényi entropies, exhibit pronounced non-monotonic or zigzag behavior, leading to low correlation coefficients. Such sensitivity likely reflects the delicate local variations in electron density within distorted clusters, indicating that not all ITA quantities are suitable descriptors for structural frustration.

A striking contrast again appears in the $\left(HF{)}_{n}\right.$ cluster: all ITA descriptors, not just the four robust ones, exhibit excellent linear correlations with the energy frustration. As before, this is attributed to the structural simplicity and uniformity of HF clusters [81].

**Figure 5:**
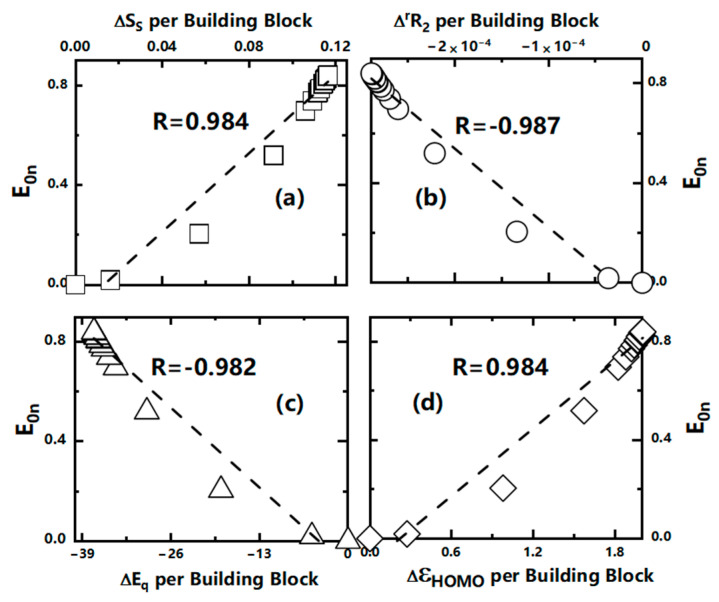
The strong linear correlations of the frustration energy per building block with two information-theoretic quantities, one energy component and one CDFT index for (HF)_n_: (**a**) Shannon entropy S_S_; (**b**) relative Rényi entropy ^r^R_2_; (**c**) quantum energy E_q_; (**d**) highest occupied molecular orbital HOMO. Units of the E_0n_ and ΔE_q_ are in kcal/mol.

Figure 5 highlights four representative linear correlations for $\left(HF{)}_{n}\right.$: two ITA descriptors (e.g., $S_{S}$ and R2r), one energy component ($E_{q}$), and one CDFT descriptor ($\varepsilon_{HOMO}$). These near-perfect linear relationships confirm that for highly uniform systems, frustration is encoded consistently across density-based descriptors, energy components, and conceptual reactivity indices.

### 4.5. Overall Perspective and Discussion

Across all four molecular systems, consistent patterns emerge. First, energy decomposition and CDFT descriptors reliably track frustration, with high and systematically interpretable correlations. Secondly, ITA descriptors provide complementary insights, but only a subset ($S_{S}$, $S_{GBP}$, $R_{2}$, and $G_{3}$) robustly reflects frustrated behavior. Thirdly, homogeneous systems with simple interaction motifs, such as $\left(HF{)}_{n}\right.$, display exceptionally coherent frustration behavior across all descriptors. Finally, more complex hydrogen-bond networks, such as $\left(H_{2}O{)}_{n}\right.$ and the charged clusters, produce descriptor-dependent variability, reflecting their richer structural flexibility.

Altogether, the results demonstrate that frustration, which is defined energetically as deviation from isolated-state structures, is encoded in multiple layers of density-based information. The combination of energy components, CDFT descriptors, and selected ITA measures provides a multi-perspective and quantitatively consistent description of the origin and nature of frustration in molecular complexes.

To further interpret these findings, it is helpful to examine why certain descriptors from the three frameworks succeed in capturing frustration while others do not. The differing performance of descriptors across the three density-based frameworks can be rationalized by considering how each responds to structural distortions that give rise to frustration. Descriptors that track global changes in electron density—such as the electrostatic and exchange–correlation energies, steric energy, and all conceptual DFT indices—exhibit smooth and monotonic variation as the monomers deviate from their isolated geometries. Because frustration is fundamentally a global property arising from collective distortion, these quantities exhibit uniformly strong linear correlations with the total frustration energy. In contrast, components such as the non-interacting kinetic energy $T_{s}$ or the quantum energy $E_{q}$ include competing local contributions that respond nonlinearly to subtle bond-angle and density-curvature changes, leading to system-dependent or sign-inconsistent behavior. A similar pattern emerges within the information-theoretic framework: only ITA descriptors that encode coarse-grained or distributional features of the electron density (e.g., $S_{S}$, $S_{GBP}$, $R_{2}$, and $G_{3}$) provide robust frustration fingerprints, whereas quantities dominated by local curvature or Laplacian features display irregular, non-monotonic trends.

These insights also explain why the $\left(HF{)}_{n}\right.$ clusters show exceptionally clean and consistent frustration correlations across all descriptors. With only a single structural degree of freedom and a simple, unidirectional hydrogen-bonding network, HF clusters undergo highly uniform distortions, smoothing out the local density fluctuations that undermine descriptor performance in more complex systems such as water clusters or charged aggregates. Taken together, these observations reveal that the ability of a descriptor to capture frustration depends on its sensitivity to global versus local density features. Descriptors that predominantly reflect global electron redistribution reliably encode frustrated behavior, while those overly sensitive to local density variations do not. This conceptual understanding offers practical guidance for selecting meaningful frustration descriptors in broader classes of molecular assemblies.

## 5. Conclusions

In this work, we conducted a systematic investigation of frustration in four molecular complex systems, two homogeneous clusters, $\left(H_{2}O{)}_{n}\right.\;$ and $\left(HF{)}_{n}\right.$, and two charged clusters, ${H_{3}O^{+}(H_{2}O)}_{n}$ and ${F^{-}(H_{2}O)}_{n},$ with the goal of elucidating both the quantitative characterization and the underlying origin of frustration. By employing three complementary density-based frameworks, including two total-energy decomposition schemes, global indices from conceptual density functional theory (CDFT), and a diverse set of information-theoretic quantities (ITA), we evaluated how different molecular descriptors respond to structural distortions that give rise to energetic frustration.

Our results reveal that most energy components and CDFT descriptors exhibit consistently strong linear correlations, either positive or negative, with the total frustration energy across all four systems. This demonstrates that these quantities effectively capture how individual building blocks sacrifice their optimal isolated-state stability to contribute to the formation and overall stabilization of the molecular complex. In contrast, only a subset of ITA descriptors, specifically Shannon entropy $S_{S}$, Ghosh–Berkowitz–Parr entropy $S_{GBP}$, Rényi entropy $R_{2}$, and relative Fisher information $G_{3},$ shows reliable and physically meaningful correlations with frustration. This selectivity reflects the distinct sensitivities of ITA measures to electron-density redistribution upon distortion.

A particularly noteworthy finding is that the $\left(HF{)}_{n}\right.$ system exhibits exceptional linearity for all descriptors examined, including ITA quantities. We attribute this to the simple hydrogen-bonding topology and single structural degree of freedom of the HF monomer, which enforces highly uniform distortion patterns across the cluster. This observation highlights how the complexity of intermolecular interactions influences the robustness of frustration descriptors.

Overall, our results demonstrate that frustration, as an intrinsic and emerging property of molecular assemblies, can be understood through a unified density-based lens integrating energetic, conceptual, and information-theoretic viewpoints. The insights gained here not only deepen our fundamental understanding of the nature and origin of frustration in molecular complexes but also provide useful guidance for studying frustrated behaviors in broader contexts, including conformational rearrangements, molecular recognition, supramolecular assembly, and enzyme catalysis.

## Figures and Tables

**Table 1:** Correlation coefficients (R) of the frustration energy per building block, E_0n_, with five energy components from two energy partition schemes (Equations (7) and (8)) for four different systems studied in this work.

R	(H_2_O)_n_	(HF)_n_	H_3_O^+^(H_2_O)_n_	F^−^(H_2_O)_n_
*T*_S_	−0.956	−0.982	−0.992	−0.953
*E*_e_	0.956	0.983	0.994	0.955
*E*_XC_	0.958	0.983	0.992	0.975
*E*_q_	−0.709	−0.982	−0.779	−0.991
*E*_S_	0.635	0.987	−0.936	0.643

**Table 2:** Correlation coefficients (R) of the frustration energy per building block, E_0n_, with five CDFT indices, $\epsilon_{HOMO}$, $\epsilon_{LUMO}$, chemical hardness η, chemical potential μ, and electrophilic index ω for the four different systems studied in this work.

R	(H_2_O)_n_	(HF)_n_	H_3_O^+^(H_2_O)_n_	F^−^(H_2_O)_n_
HOMO	0.914	0.984	0.962	0.536
LUMO	−0.965	−0.986	−0.999	−0.996
*η*	−0.966	−0.986	−0.993	−0.983
*ω*	0.962	0.988	0.998	0.988
*μ*	−0.952	−0.987	−0.643	−0.958

**Table 3:** Correlation coefficients (R) and the *p*-values (*p*) of the frustration energy per building block, E_0n_, with eleven information-theoretic quantities (Equations (14)–(23)) for the four different systems studied in this work.

R(*p*)	(H_2_O)_n_	(HF)_n_	H_3_O^+^(H_2_O)_n_	F^−^(H_2_O)_n_
*S*_S_	0.958 (3.3 × 10^−11^)	0.984 (7.2 × 10^−15^)	0.993 (6.2 × 10^−18^)	0.976 (1.1 × 10^−16^)
*I*_F_	0.635 (0.003)	0.987 (1.0 × 10^−15^)	−0.936 (1.4 × 10^−09^)	0.643 (0.002)
*S*_GBP_	0.952 (1.1 × 10^−10^)	0.985 (3.4 × 10^−15^)	0.904 (4.6 × 10^−08^)	0.990 (1.1 × 10^−16^)
*R*_2_	0.900 (6.5 × 10^−08^)	0.980 (4.7 × 10^−14^)	0.970 (1.5 × 10^−12^)	0.594 (0.006)
*R*_3_	−0.849 (2.3 × 10^−06^)	−0.985 (4.5 × 10^−15^)	0.853 (1.8 × 10^−06^)	−0.794 (3.0 × 10^−05^)
*I*_G_	−0.863 (9.7 × 10^−07^)	0.988 (3.4 × 10^−16^)	0.648 (0.002)	−0.952 (1.1 × 10^−10^)
^r^ *R*_2_	0.768 (7.8 × 10^−05^)	−0.987 (7.3 × 10^−16^)	−0.527 (0.017)	0.912 (2.5 × 10^−08^)
^r^ *R*_3_	−0.870 (6.2 × 10^−07^)	−0.990 (1.2 × 10^−16^)	0.549 (0.012)	−0.966 (4.2 × 10^−12^)
*G*_1_	−0.474 (0.035)	0.987 (1.3 × 10^−15^)	0.962 (1.4 × 10^−11^)	−0.222 (0.346)
*G*_2_	0.531 (0.016)	−0.987 (1.2 × 10^−15^)	−0.962 (1.3 × 10^−11^)	0.456 (0.043)
*G*_3_	−0.818 (1.0 × 10^−05^)	−0.985 (3.6 × 10^−15^)	−0.989 (2.5 × 10^−16^)	−0.991 (5.1 × 10^−17^)

## Data Availability

The data that support the findings of this study are available from the corresponding author upon reasonable request.

## Supplementary Materials

The following supporting information can be downloaded at: https://www.mdpi.com/article/10.3390/e28020213/s1, Table S1: Correlation coefficients (R) of energy frustration with 5 CDFT indices and 3 energy components obtained by different functionals and range-separation methods for (HF)n clusters. The basis set used is the identical 6-311+g(d,p) for all calculations; Table S2: Correlation coefficients (R) of energy frustration with 5 CDFT indices and 3 energy components obtained by different functionals and range-separation methods for F^−^(H_2_O)_n_ clusters. The basis set used is the identical 6-311+g(d,p) for all calculations; Table S3: Correlation coefficients (R) of energy frustration with 5 CDFT indices and 3 energy components obtained by different functionals and range-separation methods for H_3_O^+^(H_2_O)_n_ clusters. The basis set used is the identical 6-311+g(d,p) for all calculations; Table S4: Correlation coefficients (R) of energy frustration with 5 CDFT indices and 3 energy components obtained by different functionals and range-separation methods for (H_2_O)_n_ clusters. The basis set used is the identical 6-311+g(d,p) for all calculations; Table S5: Root-mean-square deviation (RMSD) and Procrustes disparity values of frustrated monomers in each cluster, with results from the M062X functional as the reference, for four different clusters with n = 20. Table S6: Correlation coefficients (R) of energy frustration with four ITA quantities for (H_2_O)_n_ clusters obtained by ωB97XD functional. Figure S1: Five CDFT indices for the neutral water clusters as a function of the number of building blocks, obtained using the ωB97XD functional: (a) ϵHOMO; (b) ϵLUMO, (c) Chemical hardness η; (d) Chemical potential μ; (e) Electrophilic index ω. Figure S2: Five CDFT indices for the protonated water clusters as a function of the number of building blocks, obtained using the M06-2X functional: (a) ϵHOMO; (b) ϵLUMO, (c) Chemical hardness η; (d) Chemical potential μ; (e) Electrophilic index ω. Figure S3: Five CDFT indices for the protonated water clusters as a function of the number of building blocks, obtained using the ωB97XD functional: (a) ϵHOMO; (b) ϵLUMO, (c) Chemical hardness η; (d) Chemical potential μ; (e) Electrophilic index ω.
