# Supplementary material for: Information-Theoretic and Conceptual Density Functional Theory Insights on Frustration in Molecular Clusters
Source: Entropy (Basel). 2026 Feb 12;28(2):213. doi: 10.3390/e28020213 (PMC12939162; doi:10.3390/e28020213)
Supplement: Supplementary file 1 [file entropy-28-00213-s001.zip › entropy-4077704-supplementary.pdf]

Supporting Information for:

Information-Theoretic and Conceptual Density Functional Theory Insights on Frustration in Molecular Clusters

Xinyue Zhao[1], Ziqing Yan[2], Lei Zeng[1], Yaqin Zheng[1], and Chunying Rong[1,*]

[1] Key Laboratory of Chemical Biology and Traditional Chinese Medicine Research (Ministry of Education of China), Hunan Normal University, Changsha 410081, China

[2] School of Biological Sciences and Technology, Beijing Forestry University, Beijing 100083, China

[*] Correspondence: rongchunying@aliyun.com

Table S1：Correlation coefficients (R) of energy frustration with 5 CDFT indices and 3 energy components obtained by different functionals and range-separation methods for $(HF)_n$ clusters. The basis set used is the identical 6-311+g(d,p) for all calculations.

	wB97XD	M062X em=gd3	B3LYP em=gd3bj	BLYP em=gd3bj	CAM-B3LYP em=gd3bj	M062X
	$(HF)_n$					
HOMO(eV)	0.983499938	0.976445826	0.983858659	0.984410758	0.98355955	**0.984**
LUMO(eV)	-0.986153864	-0.980119468	-0.986120733	-0.987050833	-0.986203997	**-0.986**
μ	-0.987658127	-0.983706556	-0.987287699	-0.988062411	-0.988013749	**-0.987**
η	-0.985447842	-0.978902244	-0.985545823	-0.986477445	-0.985438751	**-0.986**
ω	0.987457119	0.981764223	0.987508931	0.988949598	0.987604682	**0.988**
Ee	0.982298122	0.975276469	0.982407978	0.982987274	0.981938693	**0.983**
TS	-0.981636539	-0.974423222	-0.981708025	-0.982267483	-0.981112832	**-0.982**
Exc	0.982805035	0.975675965	0.98292986	0.983682311	0.982435178	**0.983**

Table S2： Correlation coefficients (R) of energy frustration with 5 CDFT indices and 3 energy components obtained by different functionals and range-separation methods for $F^-(H_2O)_n$ clusters. The basis set used is the identical 6-311+g(d,p) for all calculations.

	B3LYP em=gd3bj	CAM-B3LYP em=gd3bj	BLYP em=gd3bj	M062X em=gd3	wB97XD	M062X
	$F^-(H_2O)_n$					
HOMO(eV)	0.812855945	0.804879568	0.845368406	0.873556071	0.75777891	**0.536**
LUMO(eV)	-0.996469076	-0.995735358	-0.996820473	-0.996207256	-0.99630766	**-0.996**
μ	-0.984805425	-0.973661604	-0.993039752	-0.976483465	-0.972202659	**-0.958**
η	-0.990377481	-0.988850648	-0.991413487	-0.994722456	-0.986830865	**-0.983**
ω	0.996203218	0.992358827	0.998090792	0.992697928	0.992713842	**0.988**
Ee	0.957118336	0.950392294	0.950621833	0.981712738	0.944486428	**0.955**
KE	-0.95768544	-0.950438041	-0.953777435	-0.97963801	-0.945702137	**-0.953**
Exc	0.978769854	0.975066973	0.977469323	0.988018268	0.973850289	**0.975**

Table S3： Correlation coefficients (R) of energy frustration with 5 CDFT indices and 3 energy components obtained by different functionals and range-separation methods for $H_3O^+(H_2O)_n$ clusters. The basis set used is the identical 6-311+g(d,p) for all calculations.

	B3LYP em=gd3bj	BLYP em=gd3bj	M062X em=gd3bj	wB97XD	CAM-B3LYP em=gd3bj	M062X
	$H_3O^+(H_2O)_n$					
HOMO(eV)	0.975761548	0.974698598	0.972980995	0.979124221	0.974240715	**0.962**
LUMO(eV)	-0.999370671	-0.9991057	-0.999523349	-0.999537023	-0.999213762	**-0.999**
μ	-0.835633015	-0.921470283	-0.324444608	-0.88052568	-0.505229199	**-0.643**
η	-0.996346808	-0.997107952	-0.994617346	-0.996802762	-0.995226578	**-0.993**
ω	0.999353081	0.998969811	0.9996561	0.999514346	0.999424559	**0.998**
Ee	0.994501764	0.994298992	0.991266467	0.995145236	0.993718965	**0.994**
KE	-0.993499867	-0.993368901	-0.989227482	-0.994212607	-0.992455824	**-0.992**
Exc	0.995469053	0.994681278	0.994467789	0.99620851	0.994528112	**0.992**

Table S4: Correlation coefficients (R) of energy frustration with 5 CDFT indices and 3 energy components obtained by different functionals and range-separation methods for $(H_2O)_n$ clusters. The basis set used is the identical 6-311+g(d,p) for all calculations.

	wB97XD	M062X em=gd3	LC-BLYP	CAM-B3LYP em=gd3bj	BLYP em=gd3bj	B3LYP em=gd3bj	M062X
	$(H_2O)_n$						
HOMO(eV)	0.963431164	0.956550733	0.96045281	0.958908692	0.96329696	0.958543245	0.914
LUMO(eV)	−0.985690599	−0.983924275	−0.978157758	−0.979986083	−0.9832919	−0.981639137	−0.965
μ	−0.981507581	−0.942150237	−0.931193585	−0.958030877	−0.982750767	−0.972964591	−0.952
η	−0.983422796	−0.98227031	−0.979654282	−0.980036755	−0.981171705	−0.98018906	−0.966
ω	0.985182973	0.978351144	0.96815568	0.975587574	0.983684	0.980562223	0.962
Ee	0.977012839	0.976840737	0.972355215	0.973180541	0.972263079	0.973043851	0.956
Ts	−0.976899912	−0.976432143	−0.97190874	−0.972807236	−0.972090481	−0.972751232	−0.956
Exc	0.979128201	0.97846876	0.973657571	0.974541551	0.974402192	0.974737329	0.958

Table S5: Root-mean-square deviation (RMSD) and Procrustes disparity values of frustrated monomers in each cluster, with results from the M062X functional as the reference, for four different clusters with n=20.

	disparity(Å^2)	RMSD(Å)	disparity(Å^2)	RMSD(Å)	disparity(Å^2)	RMSD(Å)	disparity(Å^2)	RMSD(Å)
	$(H_2O)_{20}$		$H_3O^+(H_2O)_{20}$		$F^-(H_2O)_{20}$		$(HF)_{20}$	
B3LYP-D3BJ	0.000292	0.002204	0.001315	0.004533	0.002952	0.006956	0.005251	0.011457
BLYP-D3BJ	0.000463	0.002779	0.001862	0.005394	0.003351	0.007411	0.008036	0.014174
CAM-B3LYP	0.000211	0.001875	0.001117	0.004177	0.002845	0.006829	0.004008	0.01001
LC-BLYP	0.000112	0.001369	0.000773	0.003476	0.002586	0.006511	0.004152	0.010188
M062X	0.000000	0.000000	0.000000	0.000000	0.000000	0.000000	0.000000	0.000000
WB97XD	0.000135	0.001502	0.000907	0.003765	0.00257	0.006491	0.004411	0.010501

Table S6: Correlation coefficients (R) of energy frustration with four ITA quantities for $(H_2O)_n$ clusters obtained by ωB97XD functional.

	S_S	S_{GBP}	R_2	G_3
R	0.97915	0.98129	0.94416	−0.77132

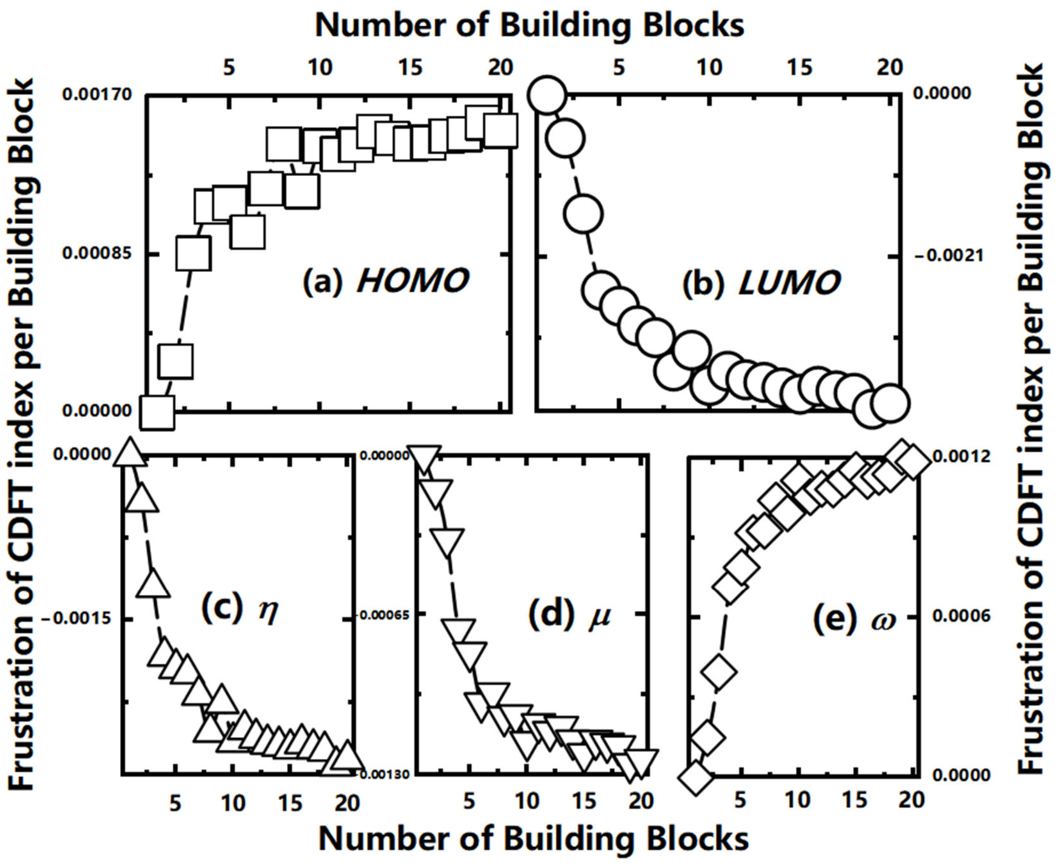

Fig. S1: Five CDFT indices for the neutral water clusters as a function of the number of building blocks, obtained using the ωB97XD functional: (a) ϵ_{HOMO}, ; (b) ϵ_{LUMO}, (c) Chemical hardness η; (d) Chemical potential μ; (e) Electrophilic index ω.

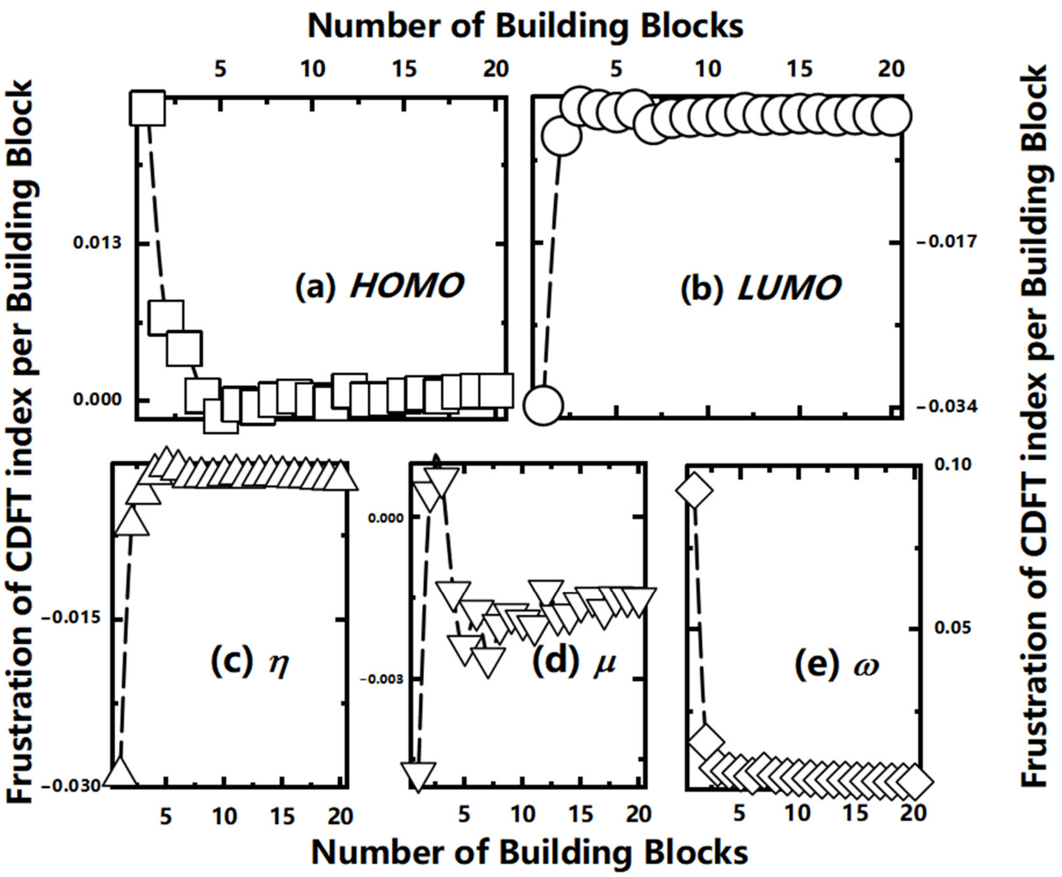

Fig. S2: Five CDFT indices for the protonated water clusters as a function of the number of building blocks, obtained using the M06-2X functional: (a) ϵ_{HOMO}, ; (b) ϵ_{LUMO}, (c) Chemical hardness η; (d) Chemical potential μ; (e) Electrophilic index ω.

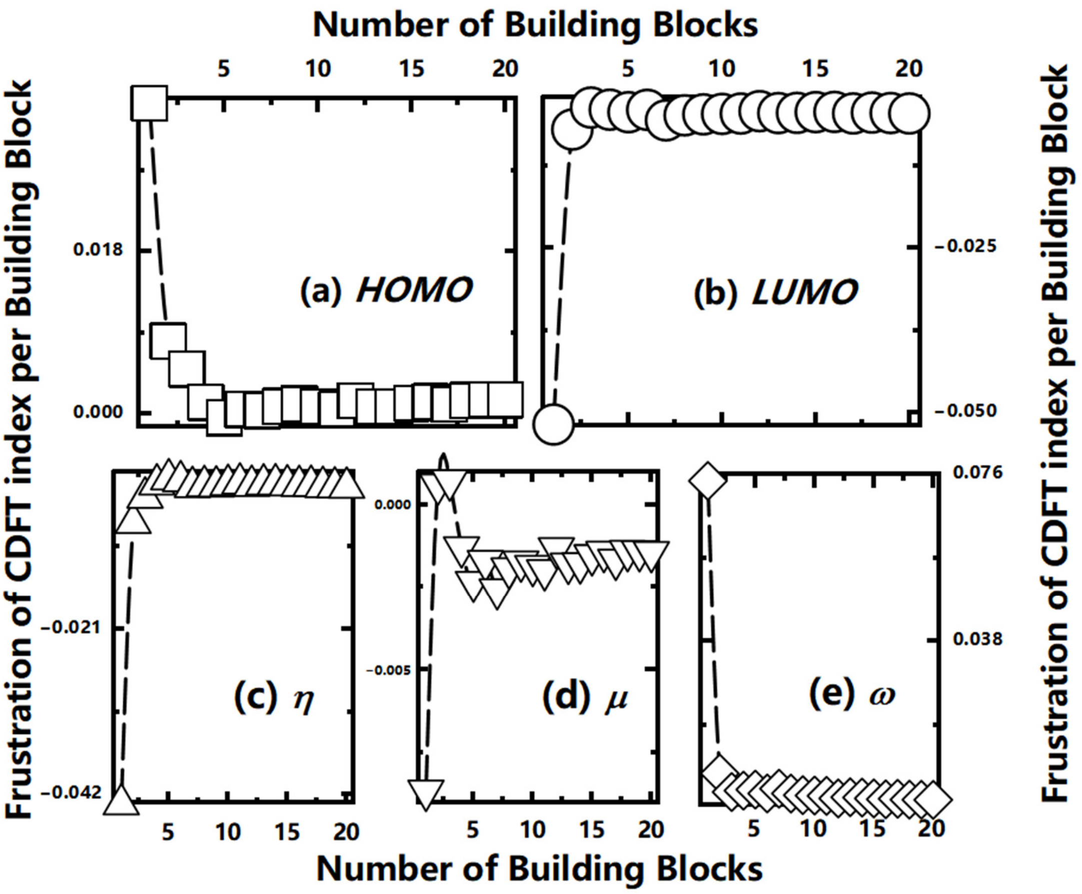

Fig. S3: Five CDFT indices for the protonated water clusters as a function of the number of building blocks, obtained using the ωB97XD functional: (a) ϵ_{HOMO}, ; (b) ϵ_{LUMO}, (c) Chemical hardness η; (d) Chemical potential μ; (e) Electrophilic index ω.
